# Perspectives and pitfalls in preserving subterranean biodiversity through protected areas

**DOI:** 10.1038/s44185-023-00035-1

**Published:** 2024-01-16

**Authors:** Stefano Mammola, Florian Altermatt, Roman Alther, Isabel R. Amorim, Raluca I. Băncilă, Paulo A. V. Borges, Traian Brad, David Brankovits, Pedro Cardoso, Francesco Cerasoli, Claire A. Chauveau, Teo Delić, Tiziana Di Lorenzo, Arnaud Faille, Cene Fišer, Jean-François Flot, Rosalina Gabriel, Diana M. P. Galassi, Laura Garzoli, Christian Griebler, Lara Konecny-Dupré, Alejandro Martínez, Nataša Mori, Veronica Nanni, Žiga Ogorelec, Susana Pallarés, Alice Salussolia, Mattia Saccò, Fabio Stoch, Ilaria Vaccarelli, Maja Zagmajster, Carina Zittra, Melissa B. Meierhofer, David Sánchez-Fernández, Florian Malard

**Affiliations:** 1 Molecular Ecology Group (MEG), Water Research Institute (IRSA), National Research Council (CNR), Corso Tonolli, 50, Pallanza, 28922 Italy; 2 Laboratory for Integrative Biodiversity Research (LIBRe), Finnish Museum of Natural History (LUOMUS), University of Helsinki, Pohjoinen Rautatiekatu 13, Helsinki, 00100 Finland; 3 NBFC, National Biodiversity Future Center, Palermo, 90133 Italy; 4 Department of Evolutionary Biology and Environmental Studies, University of Zurich, Winterthurerstrasse 190, 8057 Zurich, Switzerland; 5 Department of Aquatic Ecology, Eawag: Swiss Federal Institute of Aquatic Science and Technology, Überlandstrasse 190, 8600 Dübendorf, Switzerland; 6 cE3c—Centre for Ecology, Evolution and Environmental Changes/Azorean Biodiversity Group, CHANGE—Global Change and Sustainability Institute, School of Agricultural and Environmental Sciences, University of the Azores, Rua Capitão João d´Ávila, Pico da Urze, 9700-042 Angra do Heroísmo, Azores Portugal; 7 “Emil Racoviţă” Institute of Speleology, Department of Cluj-Napoca, Str. Clinicilor Nr. 5, 400006 Cluj-Napoca, Romania; 8 cE3c—Centre for Ecology, Evolution and Environmental Changes, CHANGE—Global Change and Sustainability Institute, University of Lisbon, Lisbon, Portugal; 9 Department of Life, Health and Environmental Sciences, University of L’Aquila, Coppito, 67100 L’Aquila, Italy; 10 Department of Organismal Biology, Université libre de Bruxelles (ULB), C.P. 160/12, Avenue F.D. Roosevelt 50, 1050 Brussels, Belgium; 11 University of Ljubljana, Biotechnical Faculty, Department of Biology, SubBioLab, Jamnikarjeva 101, SI-1000 Ljubljana, Slovenia; 12 Research Institute on Terrestrial Ecosystems of the National Research Council (IRET-CNR), Via Madonna del Piano 10, Florence, Italy; 13 Stuttgart State Museum of Natural History, Rosenstein 1, 70191 Stuttgart, Germany; 14 Interuniversity Institute of Bioinformatics in Brussels—(IB)², Brussels, Belgium; 15 Department of Functional and Evolutionary Ecology, University of Vienna, Djerassiplatz 1, 1030 Vienna, Austria; 16 Univ Lyon, Université Claude Bernard Lyon 1, CNRS, ENTPE, UMR 5023 LEHNA, F-69622 Villeurbanne, France; 17 Department of Organisms and Ecosystem Research, National Institute of Biology, Večna pot 111, SI-1000 Ljubljana, Slovenia; 18 School for Advanced Studies IUSS, Science, Technology and Society Department, 25100 Pavia, Italy; 19 Department of Zoology, University of Sevilla, Sevilla, 41012 Spain; 20 Subterranean Research and Groundwater Ecology (SuRGE) Group, Trace and Environmental DNA (TrEnD) Lab, School of Molecular and Life Sciences, Curtin University, Kent St, Bentley 6102, Perth, WA Australia; 21 Department of Chemistry, Life Sciences and Environmental Sustainability, University of Parma, Parco Area delle Scienze 11/A, 43124 Parma, Italy; 22 BatLab Finland, Finnish Museum of Natural History Luomus (LUOMUS), University of Helsinki, Pohjoinen Rautatiekatu 13, Helsinki, 00100 Finland; 23 Department of Ecology & Hydrology, University of Murcia, Murcia, 30100 Spain

**Keywords:** Biodiversity, Conservation biology

## Abstract

Subterranean ecosystems (comprising terrestrial, semi-aquatic, and aquatic components) are increasingly threatened by human activities; however, the current network of surface-protected areas is inadequate to safeguard subterranean biodiversity. Establishing protected areas for subterranean ecosystems is challenging. First, there are technical obstacles in mapping three-dimensional ecosystems with uncertain boundaries. Second, the rarity and endemism of subterranean organisms, combined with a scarcity of taxonomists, delays the accumulation of essential biodiversity knowledge. Third, establishing agreements to preserve subterranean ecosystems requires collaboration among multiple actors with often competing interests. This perspective addresses the challenges of preserving subterranean biodiversity through protected areas. Even in the face of uncertainties, we suggest it is both timely and critical to assess general criteria for subterranean biodiversity protection and implement them based on precautionary principles. To this end, we examine the current status of European protected areas and discuss solutions to improve their coverage of subterranean ecosystems.

## Introduction

It is essential to protect a considerable portion of the Earth’s biomes to slow down biodiversity loss^1,2^. Many bold post-2020 targets have been proposed to guide the establishment and maintenance of global networks of protected areas^3,4^. The Global Safety Net suggests the need to protect at least 50% of Earth’s surface to prevent further biodiversity loss and buffer the effects of climate change^5^. The European Union (EU)’s Biodiversity Strategy for 2030 seeks to create protected areas for 30% of EU’s land and sea territories—the “30 by 30” agenda^6^. Likewise, signatory countries of the United Nations Biodiversity Conference (COP 15, 7–19 December 2022, Montreal, Canada) agreed to protect 30% of the world’s land, coastal areas, and oceans by 2030. These are just a few examples among many.

**Fig. 1:**
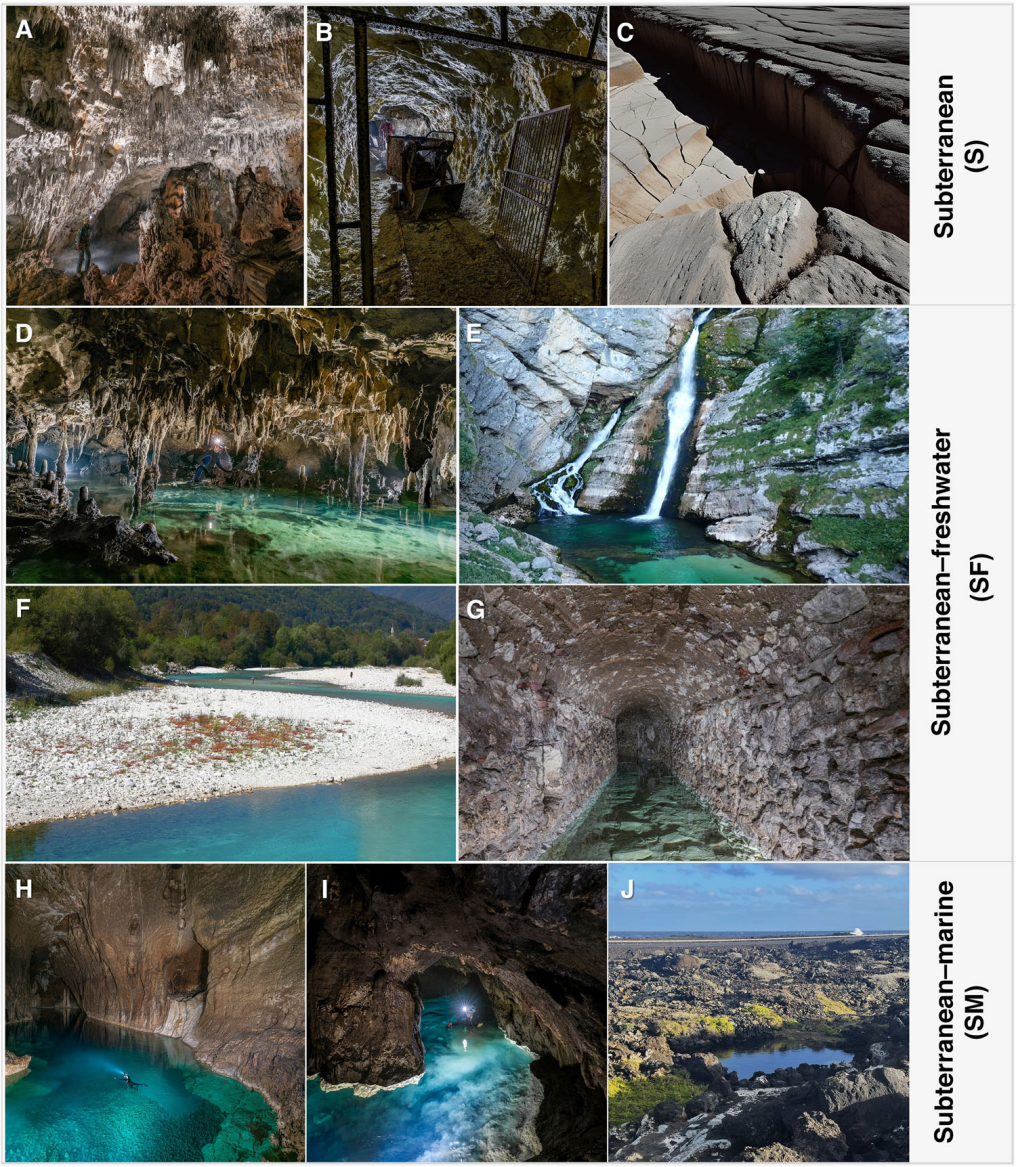
The breadth of subterranean habitats considered in this perspective. The overarching classification (Subterranean [S], Subterranean–Freshwater [SF], Subterranean–Marine [SM]) is based on ref. ^75^. **A** Terrestrial caves in different substrates (e.g., karst, lava, ice, salt); **B** Artificial subterranean habitats (e.g., mines, bunkers, blockhouses, transport tunnels, tombs); **C** Shallow and deep fissured systems; **D** Aquifers and groundwaters (e.g., subterranean lakes, rivers, ponds); **E**, **F** Springs, wells, and other surface-subterranean ecotones (e.g., voids within vadose zone in karstic and fissured aquifers, interstitial habitats such as hyporheic zone); **G** Artificial aquatic subterranean habitats (e.g., tanks, aqueducts, water pipes); **H** Marine caves, hosting coastal pools and subterranean voids connected to marine waters; **I**, **J** Anchialine caves and pools contain tidally influenced water bodies where fresh, brackish, and salt waters mix through subterranean connections between the sea and the groundwater. Anchialine pools are also exposed to open air and sunlight. Photo credits: **A**, **B**, **D**, **G**–**I** uPIX Fotografia Ipogea; **C**, **E**, **F** Ilaria Vaccarelli; **J** David Brankovits.

As we endeavor to expand the coverage of protected areas globally, we face the challenge of incorporating ecosystems with significant knowledge gaps, especially regarding their biodiversity, into protected area plans. Subterranean ecosystems are a quintessential—though not unique—example of how a lack of direct habitat accessibility, coupled with several research impediments, hampers our understanding of biodiversity^7–9^ and the implementation of evidence-based conservation measures^10^. We use the term ‘subterranean’ to refer to the extensive network of interconnected underground habitats of varying sizes (ranging from small voids to large cave chambers), substates (from unconsolidated sediments to consolidated rocks), and depths (spanning from shallow to very deep). The diversity of these environments is illustrated in Fig. 1. Except for large cavities and subterranean chambers where humans can enter, most subterranean habitats are only indirectly accessible, and their boundaries are only partly known^11–14^. Furthermore, subterranean organisms are often difficult-to-detect, numerically rare, and exhibit narrow distribution ranges, complicating biodiversity inventorying^7,8^. Consequently, data on subterranean biota tend to be biased towards caves, riddled with extensive geographic and taxonomic gaps, and scattered across a myriad of disconnected publications, datasets, and personal collections—most of which are not openly available or even lost.

Despite this incomplete and sparse knowledge, subterranean ecosystems require ample protection as they, directly and indirectly, support other ecosystems (e.g., groundwater-fed springs)^15^, are highly biodiverse^16^, and deliver numerous ecosystem services^17^. Given the aforementioned reasons and others, we cannot afford to delay the inclusion of subterranean ecosystems further in general conservation policies and agendas^18–21^. Subterranean ecosystems are increasingly threatened (Box 1), and yet, even in Europe, they remain largely uncovered by the existing network of protected areas^22^. Furthermore, with very few exceptions, decision-makers continue postponing political actions to manage these ecosystems. We believe that, even in the face of uncertainties, it is both timely and critical to assess general principles for subterranean biodiversity protection and implement them based on precautionary principles.

To this end, we established a consortium of researchers working with various subterranean ecosystems and taxonomic groups, which the aim to foster the conservation of subterranean biodiversity throughout Europe. This consortium devised into project DarCo (see “Acknowledgments” for details), whose main objective is to collate the best available data on European subterranean biodiversity and use this knowledge to promote studies on subterranean biota, identify threats, and improve the representativeness of subterranean ecosystems into the Natura 2000 network of protected areas. In this perspective, we aim to examine the major challenges associated with designing and managing subterranean protected areas. Focusing on Europe as a case study, we discuss impediments and knowledge gaps, and advance solutions to overcome them.

Box 1. Main human pressures affecting subterranean ecosystems Subterranean ecosystems are threatened by human activities at the surface (indirect impacts) and underground (direct impacts), which may affect abiotic conditions, biodiversity, and ultimately ecological functioning and the delivery of ecosystem services^76^. While the magnitude and relevance of direct versus indirect effects have been evaluated for surface freshwater systems^77,78^, these have rarely been quantified in subterranean systems beyond local case studies, with expert opinion being the only available source of information at regional to global scales^79^ (Figure Box).

Subterranean ecosystems are foremost susceptible to indirect pressures due to their connection with surface habitats and soils (e.g., through percolation of waters in crevices, wells, fractures, sinkholes, and interstitial pore spaces). Land-use change (e.g., deforestation, urbanization, agriculture, pasture, industry) burdens subterranean habitats underneath, such as by altering climatic conditions (e.g., increased heat, desertification;^80–82^) and by causing various forms of physical (e.g., siltation^83,84^, microplastics^85,86^) and chemical pollution (e.g., persistent pollutants, antibiotics, organic loads^87–89^).

Additionally, several human activities directly impact subterranean ecosystems, leading to severe and irreversible changes, albeit often localized in space and/or time. Infrastructure development (e.g., tunnel drilling, hydro-engineering, touristic facilities in show caves), extraction of rocky materials (e.g., opencast mining, quarrying), and groundwater abduction all result in habitat loss and degradation^80,90^. Furthermore, human visitation of subterranean systems, whether for scientific and caving activities or mass tourism in show caves, causes local impacts such as trampling, disturbance to the fauna, alterations to local climatic conditions, and introduction of organic materials, non-native invasive species, and pathogens^91–94^.

Finally, the combination of these regional impacts with global climate change might result in additive, synergistic, or antagonistic effects^95^. While the specifics remain debated, a recent meta-analysis linked multiple biological effects to climate change, with varying magnitudes depending on the type of subterranean habitats and ecological specialization of taxa^96^.

**Figure Box**. Examples of indirect (i.e., occurring at the surface) and direct (occurring underground) impacts on subterranean ecosystems. Color intensity (light to dark purple) reflects the relative importance of each threat according to expert opinion, based on ref. ^79^.
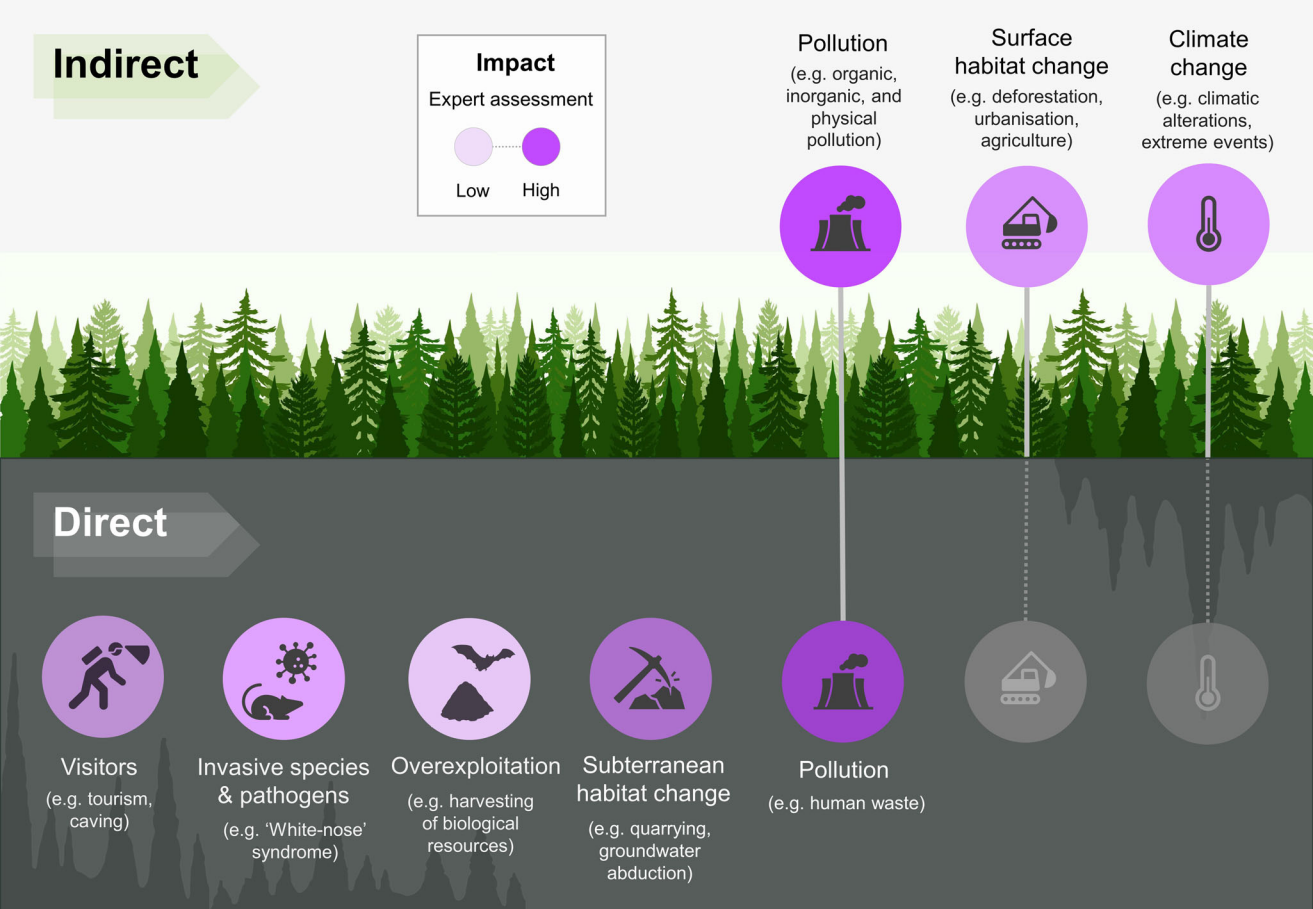

## Status of subterranean ecosystems protection

Area-based protection of subterranean ecosystems is deficient globally. Only 6.9% of karstic and volcanic landscapes bearing subterranean habitats overlap with around 17% of the terrestrial and inland waters surface that is covered by protected areas^18^. Furthermore, an estimated 85% of protected areas overlapping with aquifers do not include their catchment boundaries^23^. Importantly, most subterranean habitats receive protection primarily due to their location beneath areas designated for the conservation of surface species or habitats, without explicit consideration for their vertical and 3-dimensional nature (Box 2).

**Fig. 2:**
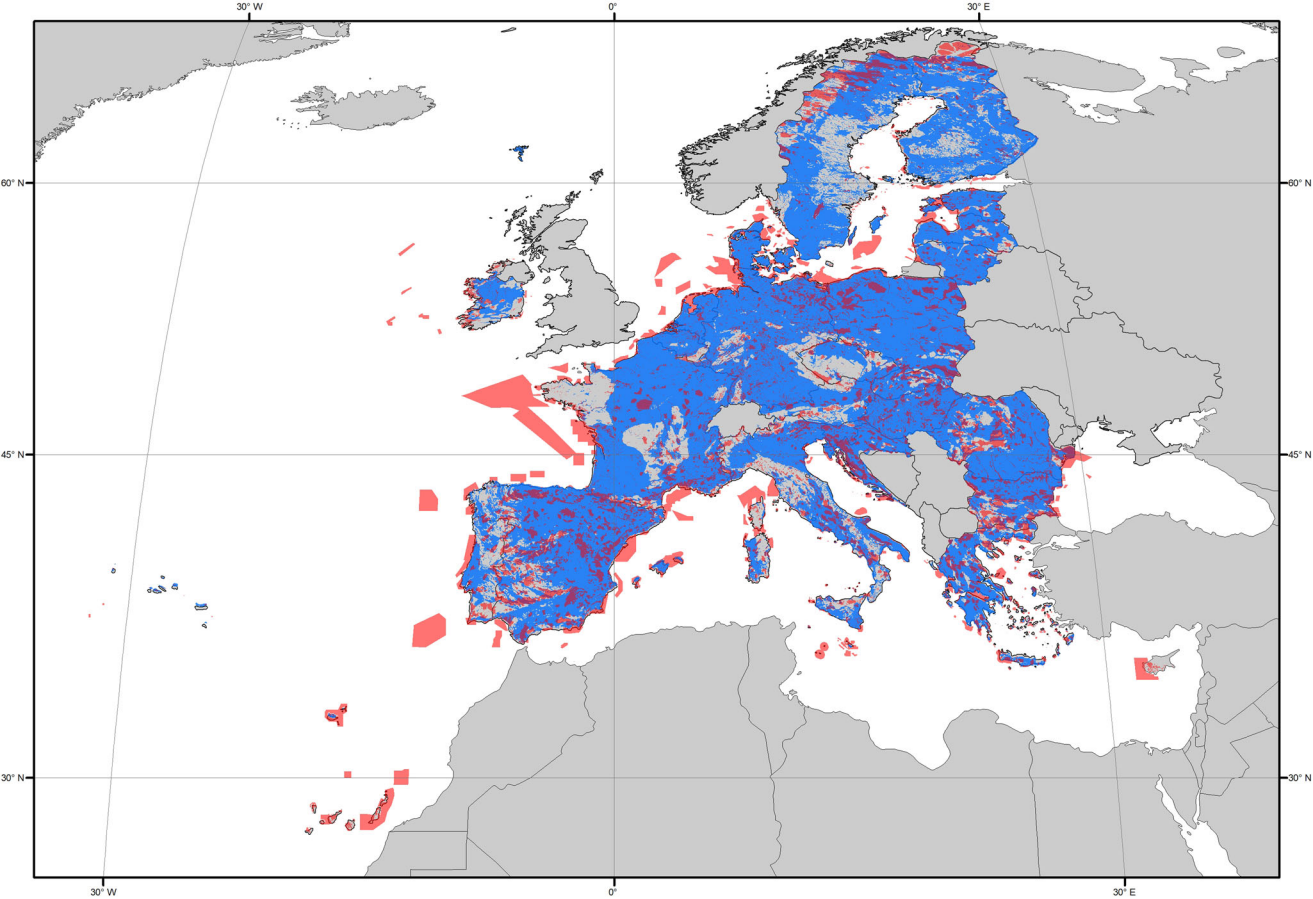
The extent of (indirect) subterranean ecosystems protection across the European Union. The map shows the European Union surface covered by the Natura 2000 network of protected areas (orange) overlaid on areas that have subterranean habitats (blue). Methods and data sources to generate the map and associated analyses are available in Supplementary Text 1.

The EU fares better than other regions with regard to subterranean protected areas, thanks to the Natura 2000 network. This is the largest transnational coordinated network of areas of conservation in the world, aiming to preserve Europe’s most valuable and threatened species and habitats as defined in the annexes of the Habitats and Birds Directives (Council Directives 92/43/EEC and 2009/147/EC). Currently, about 18% of the EU’s land area and 8% of its marine territory are covered by the Natura 2000 network^24^. According to our estimations (Supplementary Text 1), 21.84% of EU subterranean habitats are indirectly covered by the Natura 2000 network, in that they overlap with protected areas set at the surface (Fig. 2). Furthermore, the Habitats Directive lists a few specialized subterranean species [e.g., the mussel *Congeria kusceri* (Bole) and the snail *Paladilhia hungarica* Soos, the beetles *Leptodirus hochenwarti* Schmidt, *Duvalius gebhardti* (Bokor), and *Duvalius hungaricus* (Csiki), the olm *Proteus anguinus* Laurenti], several facultative subterranean species (e.g., all species of subterranean-roosting bats and all cave salamanders of the genus *Speleomantes*), and three subterranean habitat types (Table 1) in its annexes. Since the onset of the Habitats Directive, 2407 Natura 2000 sites (8.9% of total sites) were specifically established targeting these subterranean habitat types (Table 1).

**Table 1:** Number of Natura 2000 sites established for the direct protection of subterranean habitats across the European Union.

Code	Designation	*N* of sites	%
H8310	Caves not open to the public	1999	7.40
H8320	Fields of lava and natural excavations	79	0.29
H8330	Submerged or partially submerged sea caves	434	1.61
Total *	2407	8.90	

^*^Some Natura 2000 sites have been designed to cover multiple subterranean habitats, so the total number of sites (and associated percentage) is not the sum of the column “*N* of sites”.

Numbers in the percentage column (%) are calculated out of 27,027 total Natura 2000 sites.

Notwithstanding these positive trends, the protection of subterranean biodiversity remains deficient in most areas^22,25^. As for aquatic subterranean habitats, indirect protection primarily concentrates on chemical threshold values set by EU regulations (e.g., the Groundwater [2006/118/EC], Water Framework [2000/60/EC], Environmental Quality Standards [2008/105/EC], and the recast Drinking Water [2020/2184/EC] directives) to provide safe drinking water for human consumption. For groundwaters, attaining a good status entails meeting specific standards for water quantity and quality, often through the establishment of Groundwater Drinking Water Protected Areas and regulation of water abstraction. While these directives represent progress in integrating subterranean ecosystems within the same river basins under a common protection umbrella, they disregard biodiversity, ecosystem processes, and the ecological interconnections between terrestrial and aquatic subterranean compartments. Hence, it would be important to complement the current resource-oriented protection of subterranean resources with an ecosystem-oriented perspective.

Subterranean organisms and their habitat—rocks, sediments, and waters—form diverse subterranean ecosystems. While microorganisms and animals depend on certain abiotic conditions (e.g., temperature ranges, chemical composition of rocks and water) for their survival and reproduction, they, in turn, modify their abiotic environment through a range of bioengineering activities^26,27^. In doing so, they control important services provided to humans by subterranean ecosystems, such as drinking water supply. Policy and decision-makers need to recognize the ecological dimension of groundwaters and terrestrial subterranean habitats, thereby ensuring the preservation of subterranean biodiversity and ecosystem services for future generations.

Box 2. The challenge of 3-dimensionality Strategic plans for protected area designation usually include area-based targets (e.g., protecting a certain percentage of a given region). This approach, however, fails to account for the 3-dimensional nature of most habitats—their height, volume, and structure. Three-dimensionality is an essential spatial feature within diverse ecological systems^97,98^, including forest canopies^99^, freshwaters^100,101^, marine environments^102,103^, urban ecosystems^104^, the aerospace^105,106^, and diverse subterranean ecosystems^107–109^. In all these systems, organisms fulfill different aspects of their life cycles along the vertical dimension (e.g., reproduction, foraging, dispersal). However, 3-dimensionality has not yet obtained the recognition it deserves in the context of protected area targets and conservation agendas. This is problematic because omitting the vertical dimension in protected areas designation and management may lead to dreadful ecological impacts. For instance, overexploitation of groundwater resources within the Spanish Doñana National Park to supply agriculture, urbanization, and tourism has led to increasing desiccation and reduced flooding extent within the network of Mediterranean Temporary Ponds characterizing the area^110^, menacing the integrity of the Natura 2000 priority habitat and the status of Doñana as a UNESCO World Heritage Site. On the other hand, explicitly protecting subterranean ecosystems through *ad hoc* management protocols has proven to favor population recovery of endangered species, such as for the Tumbling Creek Cavesnail (*Antrobia culveri* Hubricht) in the USA^111^.

We foresee two main approaches to factor in the 3-dimensionality of subterranean ecosystems in protected area designation. A first, indirect approach relies on the protection of large buffering areas at the surface to preserve subterranean habitats underneath and maintain essential ecological processes. This stems from the understanding that subterranean biodiversity is strongly affected by above-ground processes (e.g., recharge events through rainfall^112,113^, land-use change^114^, climate change^95,96^), and that alterations at the surface can trickle down and impact the subterranean levels (Box 1). For this “buffering” approach to work effectively, it is important to test the extent to which surface-protected areas benefit the ecosystems underneath^10,115^.

A second, direct approach involves understanding and modeling the abiotic and biotic features of subterranean habitats along the vertical dimension. Previous technical gaps hampering the implementation of 3-dimensional modeling in conservation biology have been relaxed by outstanding technological advancements in laser scanning^116,117^, remote sensing^118^, and ecological modeling^119^. Nowadays, information on a representative portion of the vertical dimension of the subterranean environmental matrix can be obtained through a suite of sampling techniques targeting abiotic (e.g., terrestrial laser scanning) and biotic (e.g., pitfall traps, hand collecting, environmental DNA) features^120–122^. The obtained data could then be assembled in sets of stacked 2-dimensional gridded surfaces summarizing environmental and biotic heterogeneity of each vertical stratum (as routinely implemented for marine habitats^119^) or 3-dimensional point clouds. The analysis of such data would result in ecological models that more accurately capture the spatial variability in biodiversity features within subterranean habitats. Further, structured sampling protocols emphasizing the vertical dimension could provide data able to better represent the multivariate niche of subterranean species, describe the spatial arrangement of subterranean communities, and ultimately devise spatially explicit conservation strategies (e.g., forbidding tourism and limiting research activities in cave sectors hosting sensitive species; designing protected areas on the surface that are hydrologically linked to sensitive alluvial subterranean areas).

## Biodiversity data and surrogate variables

Identifying suitable sites for establishing protected areas involves delicate trade-offs between scientific knowledge (spatio-temporal distribution of biodiversity and human pressures) and short- and long-term socio-economic interests (political and societal needs). In essence, conservation practitioners aim to minimize costs and the total area dedicated to conservation while maximizing biodiversity protection across various facets—taxonomic, functional, and phylogenetic diversity, or even emergent properties like the delivery of ecosystem services. Hence, practitioners require high-quality biodiversity data as the foremost ingredient to develop cost-effective plans for designating protected areas.

As far as subterranean ecosystems are concerned, accumulation of high-resolution biodiversity data has progressed at a slow pace owing to technical obstacles in mapping and exploring subterranean ecosystems and intrinsic biological characteristics of subterranean organisms, including their rarity and high levels of endemism. Subterranean ecosystem boundaries are often unknown or inexact due to the existence of difficult-to-define transitional areas^28–30^. Subterranean organisms typically reside in hardly accessible networks of millimetric voids and fractures that extend below the surface down to aquicludes, aquifers, or the bedrock, rather than in large and accessible cavities. The scarcity of taxonomists working on subterranean taxa and the high frequency of cryptic species^31,32^ further complicate biodiversity inventories. As a corollary, understanding of human impacts on this biodiversity is also limited (Box 1).

While emphasizing the importance of expanding basic knowledge about subterranean biodiversity, we foresee two main approaches to avoid postponing conservation decisions. One solution is to focus prioritization assessments on the few taxonomic groups for which high-quality data are available, either because there has been a long tradition of studies (e.g., for cave-roosting bats^33^) or due to greater local knowledge often driven by the scientific interest of individual taxonomists (e.g., for harpacticoid crustaceans in Southern Europe^34^). While this approach should lead to some level of ecosystem protection, there is a main limitation associated with it. The ‘umbrella protection’ effect provided by a specific taxonomic group can be limited^35,36^. For example, a recent analysis showed that the conservation needs of cave-roosting bats only partially overlap with those of other subterranean ecosystem components^36^. Hence, for this approach to work, one would need to study correlations between the distribution of different taxonomic groups to identify the most suitable ‘biological indicators’ for prioritization exercises (see ref. ^35^. for an example with groundwater fauna). Otherwise, there is the risk that a biodiversity hotspot identified for a target taxon may not be such for other taxa.

A complementary solution, likely to work best at broader spatial scales, is to use environmental diversity as a surrogate of biodiversity for obtaining spatially continuous biodiversity data^37,38^. Over the last twenty years, subterranean ecologists have gained much experience in predicting the distribution of individual species^39^, the richness of communities^35,40^, or the range size of species^41^. They identified key environmental surrogates of subterranean biodiversity over a range of spatial scales^42,43^. In Europe, for example, the regional species richness of terrestrial and aquatic subterranean communities peaks in areas of high surface productivity and habitat heterogeneity that have not been affected by cold or arid historical events^40,44^. Furthermore, Quaternary climate oscillations have caused a clear pattern of decreasing species’ range size and increasing spatial turnover of communities with decreasing latitude in Europe^41^. A finer scales, the size of voids available to organisms (e.g., from small pores between sand grains to large cavities^45^), their interconnectedness, and their connectivity to the surface environment are key drivers of the richness and composition of local subterranean communities^46^, implying the need to protect distinct habitats within regions. Likewise, some geological formations, with certain ages or subject to particular physical-chemical phenomena, are known to be more prone to exhibit specific environmental conditions leading to species-rich subterranean assemblages^47,48^. Once the relation between environmental variables and the presence of some species (or functional groups^49^) is established, it might help in the designation of priority areas under a probabilistic framework.

## From theory to practice

Conservation stakeholders typically need conservation targets—in our case, subterranean protected areas to be established in the context of the “30 by 30” agenda. These targets need to be expressed in a measurable and clearly understandable way (e.g., maps showing conservation targets using color gradients). Unfortunately, even when conservation targets are clearly identified^33,34,50–53^, it may be impossible to address all of them, thereby requiring the use of complementarity approaches for identifying the most pressing conservation issues (e.g., using the concept of irreplaceability^54^). First and foremost, the overlap between landscapes with known subterranean habitats and areas inhabited by people should be quantified to inspire discussions about realistic goals for protected area coverage. This is critical given that globally, more than a billion people live on karst^55^, with an estimated 25% of these people located solely in Europe. Furthermore, the ‘optimal’ protection of biodiversity should extend well beyond political borders, as these are only recognized by humans. Indeed, aquifers and karst patches are often transnational^23,56^, leading to logistic and legal challenges hindering effective protection^57^. Ultimately, pinpointing conservation targets is a complicated process that requires intersecting biodiversity data (biodiversity targets), landscape features (e.g., aquifers), estimates of ecosystem vulnerability, and actual anthropic pressures to designate areas showing risks of major biodiversity loss, and embedding this consensus information into the actual territory and existing international legal framework^58^.

Once priority areas for protection have been targeted, the effective establishment of subterranean protected areas should happen after consultation among multiple actors, including researchers, policymakers, and, above all, the agencies that have control of the land through ownership or legislation. This inclusive approach increases the likelihood of adoption and benefits for everyone^59^. Although simple to write, successfully engaging a diverse range of actors with often disparate perceptions and interests is far from trivial, especially when navigating conflicts of different stakeholders^60^. Failure to include some parties and their needs may result in sabotage, political manipulation, public discontent (e.g., human-wildlife conflicts, restricted resource usage, displacement of people), and ultimately the failure of conservation efforts^61,62^. This is also applicable to subterranean ecosystems, where successful conservation outcomes are often achieved through close cooperation among conservation scientists, the media, the public, and decision makers^63^ (see Box 3 for an example). Similar integrated actions are especially needed in the context of new and emerging threats and uses of groundwater ecosystems, such as changes in hydrology due to climate change or novel uses of groundwater ecosystems for heat storage or as a novel renewable energy source^64,65^.

A well-planned narrative can positively impact public support for wildlife conservation policies^63,66,67^. The development of action plans and agreements to preserve subterranean ecosystems requires the use of common (underground!) vocabulary by the transnational actors involved, i.e., steering away from scientific jargon^68^, to ensure a broad understanding and collective agreement. It requires identifying principles and “boundary objects” that promote participation and accountability across disciplines (e.g., ecology and hydrogeology), between scientists and decision makers, and between micro- and macro-entities and authorities (EU, member states, and regions), as the subterranean realm transcends administrative boundaries^57^. Furthermore, protected areas need to evolve to withstand the effects of time, including management changes (downsizing, degazetting, variability in budget and staff^69,70^), climate change, and population growth^71,72^.

As part of future research initiatives towards subterranean biodiversity conservation, sharing of credible, legitimate, and salient information by researchers and stakeholders will be key to successfully transferring scientific and technical knowledge to decision-making and implementation of protected areas^73^. To bolster scientific credibility, future research initiatives should assemble international and diverse panels of experts in the field of subterranean biology and conservation sciences. These initiatives should promote their legitimacy by engaging with multiple stakeholders—from conservation-makers to managers, speleological groups, and users of subterranean resources—to account for their concerns and perspectives in designing subterranean protected areas. It is crucial to deliver salient information to decision makers by combining data on multiple facets of subterranean biodiversity (e.g., taxonomic, phylogenetic, and functional diversity), aquifer intrinsic vulnerability, groundwater resource use, and anthropogenic pressure. Furthermore, future initiatives must integrate these data into a systematic conservation planning model and use it as a “boundary object” through which they are able to explore networks of protected areas that best address the concerns of different actors. Effectively protecting biodiversity and functioning of subterranean ecosystems while meeting human development is a difficult but not insurmountable task, as illustrated by the recent success of subterranean protected areas in the oceanic islands of the Azores (Box 3). Yet, we acknowledge that the task is even more challenging when it is to be achieved at the scale of a densely populated continent. The DarCo initiative will aim to fulfill the above-mentioned requirements and move subterranean biodiversity conservation forward.

Box 3. A virtuous example: the establishment of subterranean protected areas in the Azores An example of the process for establishing subterranean protected areas is provided by the recent case of the Portuguese archipelago of the Azores, where subterranean protected areas were established through close cooperation between scientists, stakeholders, and policymakers. From a scientific and legal standpoint, the process involved five consecutive steps:

i. *Identification of areas needing protection*. Standardized surveys were conducted over 5 years in numerous caves in seven Azorean islands during two expeditions funded by National Geographic (1986–1989), followed by a subsequent Ph.D. student grant. Critically important was the application of standardized surveys and the participation of several taxonomists, which led to the description of 20 new subterranean species. With all the standardized data obtained, a multimetric index was created: the Importance Value for Cave Conservation (IV-CC). This index incorporated classical diversity indices for arthropod species, as well as criteria for evaluating cave geological and management features, threats, and accessibility^51^. The index suggested that larger and geologically more diverse caves have more species and higher values of IV-CC, but small caves can also have high conservation value and be irreplaceable due to the presence of narrow-endemic species and unique geological features.
ii. *Definition of a legal framework*. The Azorean Government established a task force called *Grupo para o Estudo e Salvaguarda do Património Espeleológico dos Açores* (GESPEA), which consisted of experts from the government, local speleological groups, and researchers from the University of the Azores. GESPEA is dedicated to protecting the Azores’ speleological heritage through various activities, including stakeholder consultation, underground exploration and mapping, scientific research, education, and outreach. The group currently works closely with local authorities to promote responsible cave use and management.
iii. *Writing and approval of the law*. The process of drafting and approving the legislation for subterranean protected areas in the Azores (*Decreto Legislativo Regional n*.º 10/2019/A*, de 22 de maio*) took over ten years and involved extensive stakeholders consultation.
iv. *Management of the protected areas*. Each island in the Azores appointed Park Rangers responsible for ensuring compliance with the legal framework governing the subterranean protected areas. Their role is to manage and oversee the protected areas. The management also take care of permits for carrying out scientific research in these protected areas. Permit can be requested through an online systems and are usually approved within 3 months.
v. *Implementation of monitoring schemes*. A monitoring scheme was established to ensure that the areas are being effectively managed and that emerging threats are addressed promptly. The suggested monitoring activities include establishing monitoring stations, conducting regular inspections, and assessing the overall condition of the subterranean environment.

## Conclusions

As with all conservation actions, there are potential pitfalls in establishing subterranean protected areas. It involves testing direct management solutions with limited knowledge while adapting to the changing climate (both managerial and environmental). It requires coordination among multiple actors with often highly divergent views but a common goal, all of whom may engage at different levels of the process. It means accepting the predominantly undefined nature of subterranean ecosystems and confronting their high 3-dimensionality. It implies, quite literally, operating in the dark, defining protected areas without having all the information^74^.

Regardless of these challenges, it would be irresponsible to postpone political action under the agenda that ‘more knowledge is needed’; there is no single ecosystem on Earth that we will ever fully understand. As in the successful case of Azores (Box 3), it is high time to put prioritization exercises to the scrutiny of conservation practice. What is a better place to start than within the well-established infrastructure of the Natura 2000 network and the ambitious agenda of the EU Biodiversity Strategy for 2030?

### Supplementary information

Supplementary Material
