# Supplementary material for: Perspectives and pitfalls in preserving subterranean biodiversity through protected areas
Source: NPJ Biodivers. 2024 Jan 16;3:2. doi: 10.1038/s44185-023-00035-1 (PMC11332058; doi:10.1038/s44185-023-00035-1)
Supplement: Supplementary file 1 — Supplementary Material [file 44185_2023_35_MOESM1_ESM.pdf]

SUPPLEMENTARY MATERIAL FOR

Perspectives and pitfalls in preserving subterranean biodiversity through protected areas

Mammola S., *et al.*

TABLE OF CONTENTS

Text 1. Methods for generating the map in Figure 2.

Text 1. Methods for generating the map in Figure 2.

We conducted all spatial analyses with QGIS v 3.14.0[1]. To get an area-based approximation of subterranean habitats in Europe, we used the information provided by Cornu et al. (ref. [2]) including all habitat categories from the shape file except the category “practically non-aquiferous rocks" and the world map of karstifiable rocks and aquifers (carbonates, evaporites, basalts)[3]. We dissolved these maps (to get a unique category) and clipped them by the UE27 map (see light blue surface in Figure 2). Then, we overlapped this map with the map of the Natura 2000 network of protected areas (red surface), which we downloaded from the European Environmental Agency (available at: https://ec.europa.eu/environment/nature/natura2000/data/index_en.htm; accessed on 12 June 2023).

Literature cited

1. QGIS Development Team QGIS Geographic Information System. Open Source Geospatial Foundation Project (2019). http://qgis.osgeo.org

2. Cornu, J. F., Eme, D. & Malard, F. The distribution of groundwater habitats in Europe. Hydrogeol. J. 21, 949–960 (2013).

3. Chen, Z., Goldscheider, Auler, N., Bakalowicz, M., Broda, S., Drew, D., Hartmann, J., Jiang, G., Moosdorf, N., Richts, A., Stevanovic, Z., Veni, G., Dumont, A., Aureli, A., Clos, P., Krombholz, M. World Karst Aquifer Map (WHYMAP WOKAM). BGR, IAH, KIT, UNESCO (2017).. https://doi.org/10.25928/b2.21_sfkq-r406
